# Computer-Aided Assessment of Melanocytic Lesions by Means of a Mitosis Algorithm

**DOI:** 10.3390/diagnostics12020436

**Published:** 2022-02-08

**Authors:** Bart Sturm, David Creytens, Jan Smits, Ariadne H. A. G. Ooms, Erik Eijken, Eline Kurpershoek, Heidi V. N. Küsters-Vandevelde, Carla Wauters, Willeke A. M. Blokx, Jeroen A. W. M. van der Laak

**Affiliations:** 1 Department of Pathology, Radboud University Medical Center, 6500 HB Nijmegen, The Netherlands; b.sturm@pathan.nl; 2 Pathan B.V., 3045 PM Rotterdam, The Netherlands; j.smits@pathan.nl (J.S.); a.ooms@pathan.nl (A.H.A.G.O.); e.kurpershoek@pathan.nl (E.K.); 3 Department of Pathology, Ghent University Hospital, 9000 Ghent, Belgium; david.creytens@uzgent.be; 4 Laboratory for Pathology Oost Nederland (LabPON), 7550 AM Hengelo, The Netherlands; e.eijken@labpon.nl; 5 Department of Pathology, Canisius Wilhelmina Hospital, 6500 GS Nijmegen, The Netherlands; h.kusters@cwz.nl (H.V.N.K.-V.); c.wauters@cwz.nl (C.W.); 6 Division Laboratories, Pharmacy and Biomedical Genetics, University Medical Center Utrecht, 3508 GA Utrecht, The Netherlands; W.A.M.Blokx@umcutrecht.nl; 7 Center for Medical Image Science and Visualization, Linköping University, 581 83 Linköping, Sweden

**Keywords:** WSI, computer-aided diagnosis, mitosis algorithm, melanoma, nevoid melanoma

## Abstract

An increasing number of pathology laboratories are now fully digitised, using whole slide imaging (WSI) for routine diagnostics. WSI paves the road to use artificial intelligence (AI) that will play an increasing role in computer-aided diagnosis (CAD). In melanocytic skin lesions, the presence of a dermal mitosis may be an important clue for an intermediate or a malignant lesion and may indicate worse prognosis. In this study a mitosis algorithm primarily developed for breast carcinoma is applied to melanocytic skin lesions. This study aimed to assess whether the algorithm could be used in diagnosing melanocytic lesions, and to study the added value in diagnosing melanocytic lesions in a practical setting. WSI’s of a set of hematoxylin and eosin (H&E) stained slides of 99 melanocytic lesions (35 nevi, 4 intermediate melanocytic lesions, and 60 malignant melanomas, including 10 nevoid melanomas), for which a consensus diagnosis was reached by three academic pathologists, were subjected to a mitosis algorithm based on AI. Two academic and six general pathologists specialized in dermatopathology examined the WSI cases two times, first without mitosis annotations and after a washout period of at least 2 months with mitosis annotations based on the algorithm. The algorithm indicated true mitosis in lesional cells, i.e., melanocytes, and non-lesional cells, i.e., mainly keratinocytes and inflammatory cells. A high number of false positive mitosis was indicated as well, comprising melanin pigment, sebaceous glands nuclei, and spindle cell nuclei such as stromal cells and neuroid differentiated melanocytes. All but one pathologist reported more often a dermal mitosis with the mitosis algorithm, which on a regular basis, was incorrectly attributed to mitoses from mainly inflammatory cells. The overall concordance of the pathologists with the consensus diagnosis for all cases excluding nevoid melanoma (*n* = 89) appeared to be comparable with and without the use of AI (89% vs. 90%). However, the concordance increased by using AI in nevoid melanoma cases (*n* = 10) (75% vs. 68%). This study showed that in general cases, pathologists perform similarly with the aid of a mitosis algorithm developed primarily for breast cancer. In nevoid melanoma cases, pathologists perform better with the algorithm. From this study, it can be learned that pathologists need to be aware of potential pitfalls using CAD on H&E slides, e.g., misinterpreting dermal mitoses in non-melanotic cells.

## 1. Introduction

Digital pathology is a dynamic, image-based environment that enables the acquisition, management, and interpretation of pathology information generated from a digitised glass slide, i.e., whole slide images (WSI), that can be assessed on a computer screen. Digital pathology offers all kinds of benefits, including digital archiving, consultation, and showcasing at tumour boards [1,2]. It is an innovation committed to the improvement of operational efficiency, including decreasing turn-a-round times with the reduction of laboratory expenses [2,3]. Since several WSI scanners are approved in Europe, given the European Conformity mark, in the United States of America by the Food and Drug Administration, and in Japan, by the Pharmaceuticals and Medical Devices Agency [4], enormous opportunities have arisen to analyse the sheer amount of slides with visual quantitative computer techniques, i.e., computational pathology (CP), based on machine learning (ML) [2]. CP may aid in assessing WSI for pathology diagnosis, so called computer-aided diagnosis (CAD). A range of different ML techniques are available of which in recent years, algorithms based on convolutional neural networks appear to dominate [5].

Malignant melanoma is the most lethal form of skin cancer and its prevalence varies among regions in the United States of America [6,7,8,9]. Research on CAD pathology has focused on different applications, including prostate cancer tumour detection and Gleason scoring [10,11,12,13] and breast cancer identification, grading (including assessing mitosis), hormone immunohistochemical status, and lymph node metastases [13,14,15,16,17,18,19,20,21,22,23,24]. Concerning CAD application in melanoma diagnostics, studies have been performed mainly on immunohistochemical (double) stains, e.g., phosphohistone H3, KI67, and/or MART1 [25,26]. Although diagnosing melanocytic lesion can be very challenging, CAD has not been studied extensively for melanoma pathology diagnostics on hematoxylin and eosin (H&E) stained slides. The presence and foremost the enumeration of subtle cytomorphologic and architectural features such as asymmetry of the lesion, cytological atypia, Pagetoid involvement of the epidermis, lack of maturation, presence of ulceration, and mitosis can make a major difference between a benign nevus, an intermediate lesion, or a malignant melanoma. Finding a mitosis in a melanotic cell, situated either epidermal or dermal, is of major importance as it implies the lesion might be intermediate or malignant [27]. It was shown that dermal mitoses indicate worse prognosis for survival and increased occurrence of sentinel node metastases [28,29,30,31].

In recent years, the development of computer-aided mitosis detection has increased significantly, which is partly due to publicly released training data sets for mitosis detection [32,33,34,35]. In a previous study, Tellez et al. trained a convolutional neural network (CNN) to detect individual mitotic figures in breast carcinoma WSI’s with high accuracy [18]. The current study aimed to assess if a mitosis algorithm developed for breast cancer (1) can be used in the detection of mitosis in cutaneous melanocytic tumours, and (2) can improve the accuracy of the diagnosis of melanocytic lesions in a practical setting.

## 2. Materials and Methods

### 2.1. Case Selection and Study Design

In this study we used WSI of 102 H&E stained cases from a previous study, evaluating the potential added value of z-stack scanning in diagnosing melanocytic lesions [36]. The cases were obtained from the archive of the Pathology Department of the Radboud UMC in Nijmegen, The Netherlands, and concerned 35 benign nevi, 5 intermediate lesions (so-called melanocytomas or melanocytic tumours of unknown malignant potential; MELTUMP), and 62 malignant melanomas, including 10 nevoid melanomas. The set of WSI assessed by the study pathologists contained 99 cases for which consensus could be achieved by 3 academic pathologists based on the glass slides (35 benign melanocytic lesions, 4 intermediate cases, and 60 melanomas, including 10 nevoid melanomas) [36]. The cases, scanned with a Pannoramic 250 flash II scanner (3D Histech, Budapest, Hungary), were re-randomized and submitted for evaluation to 8 pathologists that participated in the previous study (2 academic and 6 general pathologists). All WSI were assessed twice, first without and second with help of a mitosis detection algorithm, with a washout period of at least 2 months in between. The WSI cases were presented on a computer with a calibrated high resolution 4K LCD screen. Cases were offered with concise clinical information (age, gender, and location on the skin) and could be classified by the pathologists as either benign, malignant, or intermediate. For lesions classified as malignant or intermediate, the presence of dermal mitotic activity had to be reported. In addition, lesions classified as intermediate could be stratified into low risk and high risk. During the second assessment the pathologists were also asked to indicate which mitoses identified by the algorithm were helpful in making the diagnosis including cases that they classified as benign. More details on the study design are provided in our previous study [36].

### 2.2. Mitosis Algorithm

**Figure 1:**
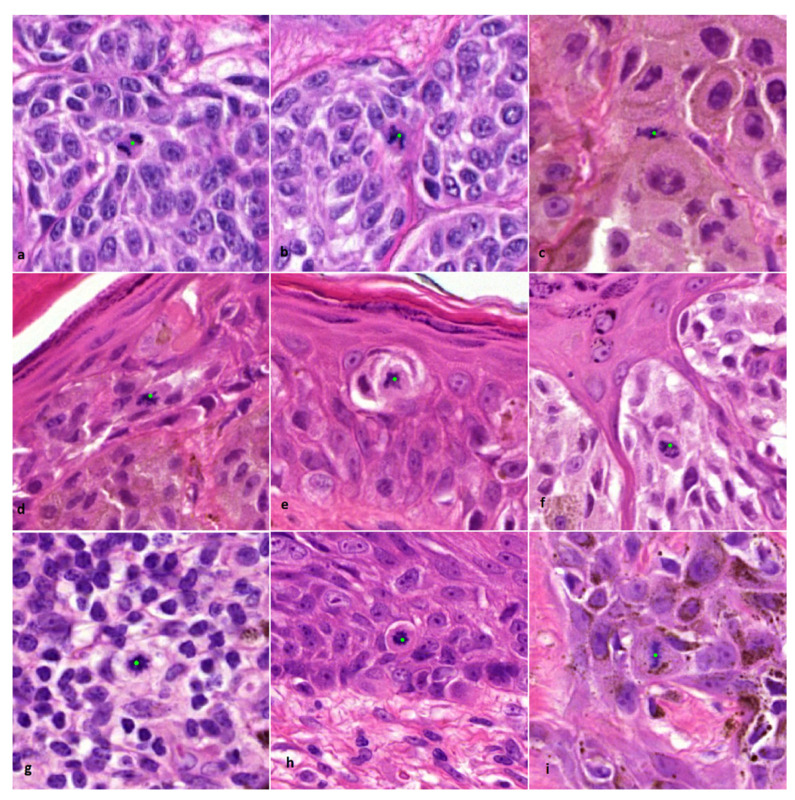
Examples of correctly indicated lesional and non-lesional mitoses by the algorithm (×800 magnification). (**a**–**c**) Dermal mitosis in a melanocyte. (**d**–**f**) Epidermal mitosis in a melanocyte. (**g**) Dermal mitosis in an inflammatory cell. (**h**,**i**) Epidermal mitosis in a (pigmented) keratinocyte. Classification of indicated mitoses and type of cell origin was performed by an experienced pathologist (BS).

**Figure 2:**
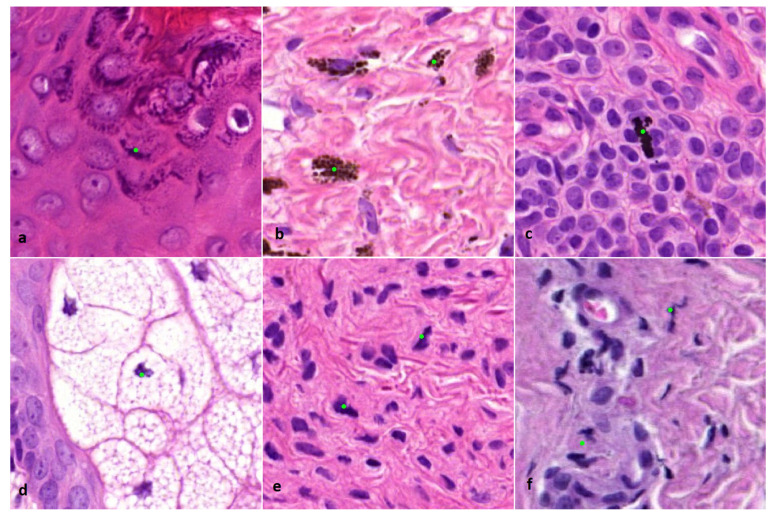
(×800 magnification). Examples of incorrectly indicated mitoses by the algorithm. (**a**) Keratin granules. (**b**) Melanin pigment. (**c**) Formalin pigment. (**d**) Nucleus of a sebaceous cell. (**e**) Spindle cell nucleus of a melanocyte. (**f**) Squeezed nucleus of a lymphocyte.

The mitosis algorithm used in this study was developed for automated detection of mitoses in breast carcinomas and is based on CNNs [18]. Therefore, in cutaneous melanocytic lesions false positive mitosis diagnoses were to be expected, because of different background stroma, colour, texture and the potential presence of melanin pigment. Before the start of the current study, a small pilot study was done to see if the algorithm was capable of detecting mitoses in 10 melanocytic cases. From these cases it was learned that mitoses could be identified correctly (Figure 1), although false positive mitoses were indicated by the algorithm as well (mostly consisting of melanin pigment, sebaceous gland nuclei, and spindle cell nuclei such as stromal cells and neuroid differentiated melanocytes) (Figure 2).

In order to assess the algorithm’s practical use in assisting pathologists to find mitoses in the current study, a selection of candidate mitoses was made (BS) before the annotated cases were offered to the participating pathologists. The selection excluded non-nucleated objects and nuclei of sebaceous glands specifically.

Application of the mitosis CNN was limited to a manually defined region of interest (ROI), using freely available ASAP software (version 1.8.1). The ROI was defined by an experienced pathologist (BS) and delineated the melanocytic lesion.

### 2.3. Statistical Analysis

For statistical analysis the four-tier scheme defined above was downsized to a three-tier system by combining high-risk and low-risk intermediate lesions, as discrimination of these lesions based on an H&E staining only without ancillary techniques is often not feasible [37]. Concordance of pathologists with the consensus diagnosis was expressed as the number and percentage of cases with identical diagnoses (in the three-tier system) for every subclass as well as overall. As an overall measure of concordance of the pathologists with the consensus diagnosis, Kappa statistics with 95% confidence intervals (CIs) were calculated. Data from our previous study was used for analysing the variation over time.

## 3. Results

In total, 2868 objects (range 1–676 per case) were detected by the algorithm in 76 cases that classified for a (candidate) mitosis. After manual selection, 825 (candidate) mitoses were retained that were annotated in 61 cases comprising mainly epidermal and dermal mitoses, i.e., lesional but also non-lesional mitoses in keratinocytes and inflammatory cells. Furthermore, spindle cell nuclei, apoptotic cells and contused nuclei of e.g., lymphocytes, were annotated as well.

The overall concordance of the pathologists with the consensus diagnosis for all cases excluding nevoid melanoma (*n* = 89) appears to be comparable with and without the use of AI (89% vs. 90%), shown in Table 1. Agreement according to Cohen’s Kappa is at least substantial with and without the mitosis algorithm, shown in Table 2, except for pathologist PATH4 reaching at least moderate agreement with the consensus diagnosis.

**Figure 3:**
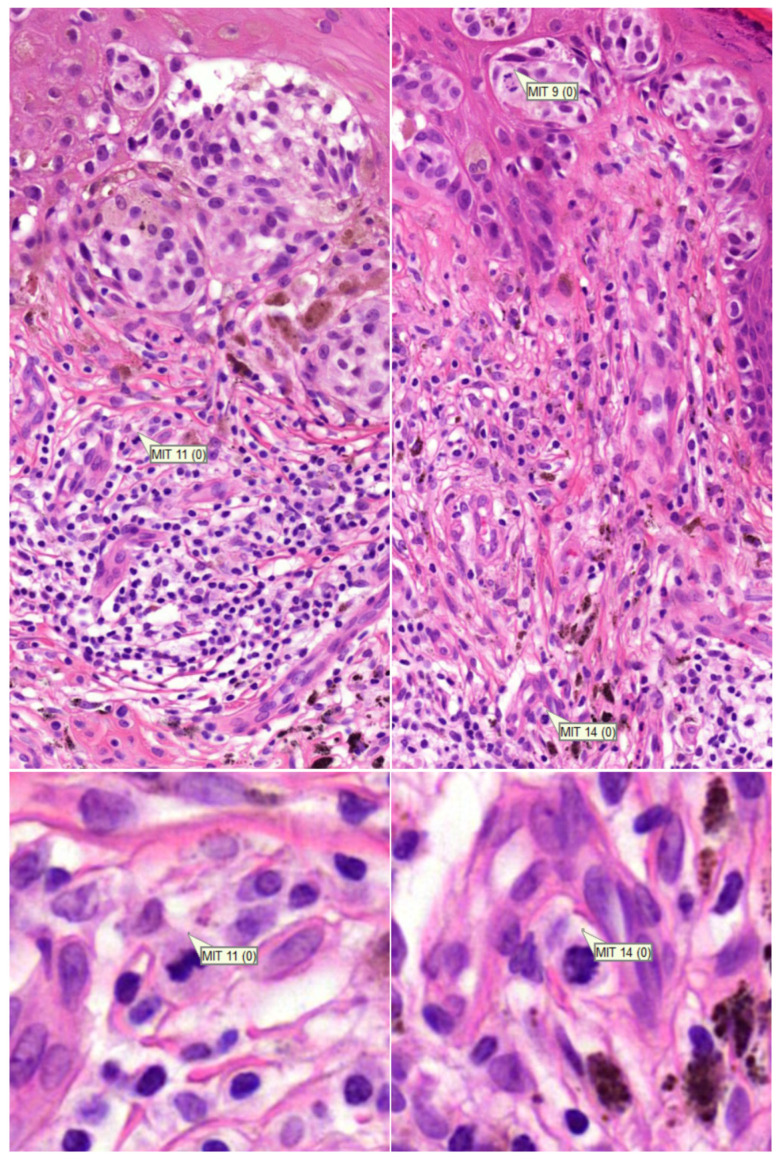
(row above ×200, row below ×800 magnification). Examples of incorrectly attributed mitoses from inflammatory cells located outside of the tumour front.

**Figure 4:**
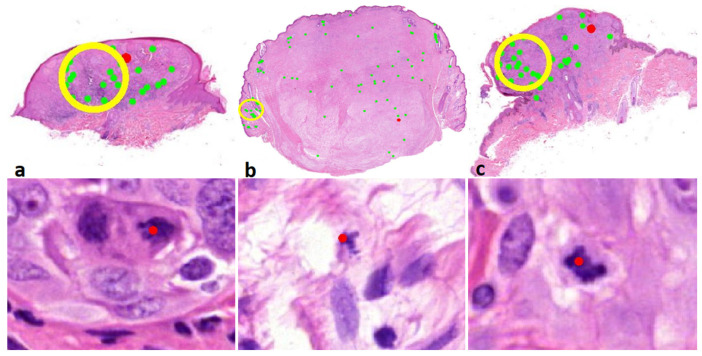
(row above ×6, row below ×800 magnification). Correct dermal mitosis discordant with the consensus. Above overview with red indicates mitosis, below a detailed view of the indicated mitosis. (**a**) Case 56; melanocytoma/intermediate lesion, high risk. (**b**) Case 73; desmoplastic melanoma. (**c**) Case 100; melanocytoma/intermediate lesion, low risk.

In Table 1 the number of cases with reported dermal mitoses are presented as well. According to the consensus diagnosis, in 28 cases out of a total of 54 malignant (excl. nevoid melanoma) plus intermediate cases, dermal mitoses were present. All pathologists excluding one academic pathologist reported more dermal mitoses with the mitosis algorithm. Three pathologists reported a substantially higher number of cases with dermal mitoses (PATH1, PATH2, and PATH4). After reviewing these cases (BS), it appeared that on a regular basis, mitoses in infiltrates nearby the tumour front were interpreted as falsely being mitoses from melanocytes (Figure 3). However, in three cases (cases 56, 73, and 100), at least one pathologist reported a dermal mitosis by means of the algorithm that was reconfirmed by the investigator (BS), and was discordant with the consensus diagnosis based on glass slides (Figure 4), although the consensus concerning the presence of a dermal mitosis was not unanimous at the time. Furthermore, pathologist PATH4 had a significant lower concordance rate with the mitosis algorithm, where the algorithm did aid in correctly changing the diagnosis from benign to malignant in six cases versus incorrectly changing the diagnosis four times. The lower concordance was mainly due to incorrectly changing the diagnosis from benign to intermediate in eight cases in which two cases of dermal mitosis was reported incorrectly with the mitosis algorithm.

**Figure 5:**
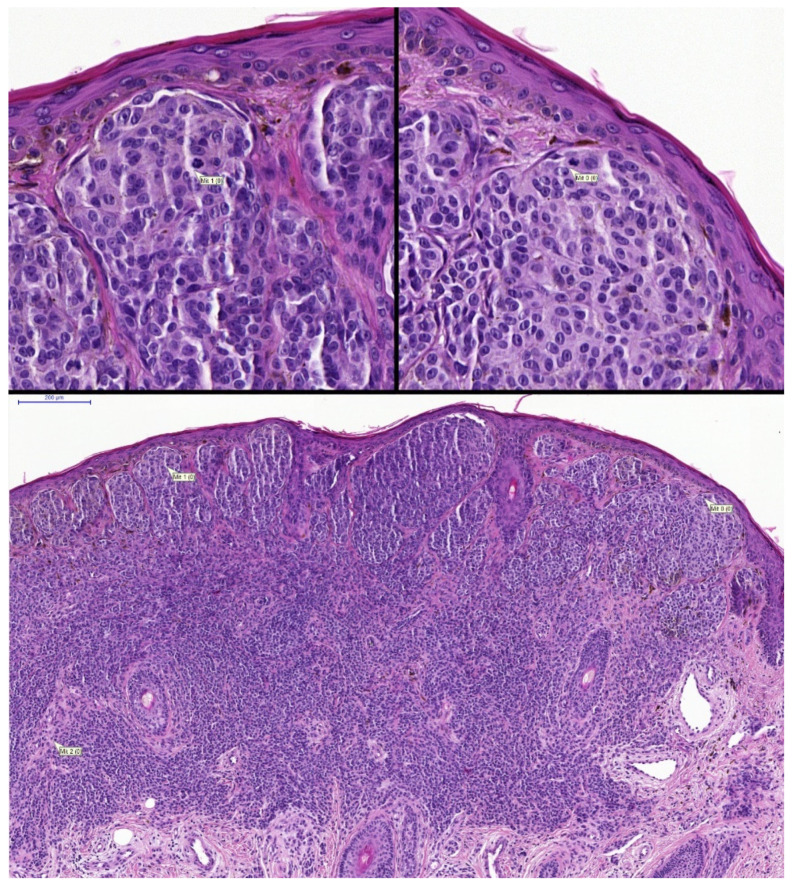
(row above ×400, row below ×100 magnification). Example of nevoid melanoma with two annotated correct lesional dermal mitoses. Case 71; two pathologists changed the diagnosis from benign to malignant with the aid of the algorithm.

In general, the pathologists appeared to have an advantage with the mitosis algorithm in the nevoid melanoma cases (*n* = 10), as shown in Table 3 and example given in Figure 5, although the number of cases with reported dermal mitosis on average kept constant with and without the mitosis algorithm, i.e., five to six. If melanoma and intermediate diagnosis were grouped together, all but one pathologist performed better with the algorithm, recognizing the nevoid melanoma cases as being at least potentially malignant.

## 4. Discussion

The application of AI in routine pathology diagnostics is on the rise. This study is the first, to the best of our knowledge, to actually use a CNN-based mitosis algorithm to aid pathologists in assessing melanocytic lesions in a routine diagnostic setting. Former studies have shown that a deep learning algorithm has the potential to improve diagnostic workflow in diagnosis i.e., nodular basal cell carcinoma, seborrheic keratosis, dermal nevus, and melanoma [38,39]. Andres et al. presented a proof-of-principle of a computer-aided staging support system for malignant melanoma [40]. Studies have shown that immunohistochemical markers i.e., PHH3, Ki-67, P16, HM45, and PRAME can aid pathologists in the diagnosis of melanoma and may reduce observer variation [26,41,42,43,44,45]. However, for pathologists it is more convenient to make all analyses, including CAD, on H&E WSI, mostly because it is less time-consuming and expected to be less expensive than immunohistochemistry in the coming years. Mitosis detection on H&E slides has been investigated thoroughly for breast cancer histopathology, where mitotic density was shown to be prognostic and is therefore part of the grading system and is of importance for patient management [19]. For melanoma diagnosis, the performances of mitosis algorithms have been tested in skin tissue with promising results [40,46]. Studies investigating tumour grading have reported that it is difficult to establish a ground truth for mitotic cells. Criteria for defining a mitosis can be given but in practice these criteria are difficult to follow in a strict manner by pathologists because of doubtful instances where subjective interpretations must be made. Misinterpretation of mitoses can occur due to similarity to, for example, apoptotic cells. As a result, a relatively large inter- and intra-observer variation in recognizing mitoses is a fact [19]. This is reflected in our previous study on the effect of z-stack scanning, where pathologists reported dermal mitoses in a range from 17 to 30 cases (*n* = 54) [36]. Nevertheless, in the current study, even without the algorithm, the range narrowed to 21–27. This may be a result of more experience with assessing WSI’s by the pathologists, since the execution of the former study was during the years 2016 and 2017.

Tabata et al. found that traditional light microscopy was measurably more accurate in detecting mitoses than WSI [4]. This may be explained because of the omission of focussing in different z-planes in WSI. Another explanation is that WSI’s are scanned on 20× objective while in microscopy assessment mitoses are mostly found on 40× objective. Although it is reported that in WSI less mitoses are detected compared to light microscopy, in our study a larger amount of dermal mitoses was reported on WSI with the aid of the mitosis algorithm. On a regular basis, these mitoses were attributed falsely by some pathologists to mitoses of inflammatory cells and, to a lesser extent, to the difficulty of interpreting mitoses as, for example in squeezed nuclei of lymphocytes, bizarre nuclei of melanomas or apoptotic cells. However, in three cases at least one pathologist reported a dermal mitosis by means of the algorithm that was discordant with the ground truth based on glass slides underpinning the potential advantage of the algorithm.

Concerning the nevoid melanoma cases, a mitosis was found sporadically with the aid of the algorithm, changing the diagnosis from benign to either intermediate or melanoma. Remarkable is the fact that only two of the eight pathologists reported more dermal mitosis in the nevoid melanoma cases with the mitosis algorithm, although it did not aid significantly in recognizing nevoid melanoma. In case 16 and 71, respectively, three and two pathologists changed their diagnosis from benign to malignant. In these two cases pathologists reported, respectively, one and four dermal mitoses to be of aid for the diagnosis. After reviewing the reported mitoses these were dermal mitoses in melanocytes (BS). On the other hand, in some nevoid melanoma cases, despite the awareness of the presence of dermal mitoses by the pathologist, these melanocytic lesions were still falsely interpreted by several pathologists as benign, reflecting the complex interpretation of this class of malignant lesions.

Finally, in this study, a high number of false positive mitotic objects were indicated by the algorithm, e.g., spindle cell nuclei of stromal and melanocytic cells and squeezed nuclei of mainly inflammatory cells, which appeared time-consuming for the pathologists to assess. These false positive objects were an expected finding as the CNN was not trained for skin tissue and melanocytic tumours, mainly due to a shortage of resources to tune the algorithm for this purpose. Although this is a limitation of our study design, we did overcome this by a manual selection of (candidate) mitoses. Nevertheless, a CNN can be optimized in finding mitoses while simultaneously discriminating similar objects that are not of interest to the pathologist. A perfect mitosis algorithm will indicate all mitoses, not limited to the cell of interest, in the study of the melanocyte. This points out another limitation of the study, that the mitosis algorithm doesn’t discriminate between a mitosis from a melanocyte, keratinocyte, lymphocyte, or other cell. Immunohistochemical double stains (PHH3, Ki-67 and/or MART1) can overcome this uncertainty, although it is costly and time-consuming. In daily practice, the cell of origin is mainly classified on the basis of the location of the mitosis, i.e., in a lesional or non-lesional area. In order to effectively aid pathologists in identifying mitosis in melanocytic lesions, an algorithm should preferentially discriminate mitosis in a melanocytic cell and omit mitoses mainly from keratinocytes and inflammatory cells, as well as difficult-to-interpret objects such as, for example, squeezed nuclei. Therefore, optimization in the differentiation of mitosis (like) objects may be one of the next objectives in CNN mitosis detection development, e.g., to discriminate areas of interest in lesional epidermis, lesional dermis, and non-lesional stroma.

## 5. Conclusions

Diagnosing melanocytic lesions is challenging and may have major implications on patient management and wellbeing. This study shows that a mitosis algorithm that was primarily developed for breast cancer can be applied to melanocytic skin lesions, although it is not applicable in a practical setting due to a high number of false positive-indicated mitoses. After a correction procedure for false positive (candidate) mitoses, it appeared that in general cases, pathologists perform similarly with the aid of a mitosis algorithm in WSI. However, pathologists perform better with the algorithm in nevoid melanoma cases, which are notoriously difficult to recognise. From this study it can be learned that pathologists need to be aware of potential pitfalls using computer-aided diagnosis on H&E slides, such as misinterpreting dermal mitosis from non-melanotic cells, i.e., mainly inflammatory cells.

## Figures and Tables

**Table 1:** Concordance as a percentage (%) of cases identical with the consensus diagnosis based on glass slides for all cases excluding nevoid melanoma (*n* = 89) and number of cases with reported dermal mitosis (#DM) concerning intermediate lesions and melanoma (excl. nevoid melanoma) (*n* = 54). ^a^ Glass; ^b^ WSI.

Pathologist	z-Stack Study [36] Glass ^a^/WSI ^b^		1st Round WSI		2nd Round WSI Algorithm	
	%	#DM	%	#DM	%	#DM
EXP1	97 ^a^	30 ^a^	94	27	91	27
EXP2	93 ^a^	22 ^a^	91	26	89	27
PATH1	89 ^b^	19 ^b^	91	25	91	44
PATH2	89 ^b^	22 ^b^	96	25	94	35
PATH3	81 ^b^	20 ^b^	87	26	92	27
PATH4	75 ^b^	17 ^b^	84	27	76	40
PATH5	84 ^b^	21 ^b^	96	25	94	29
PATH6	90 ^b^	18 ^b^	84	21	84	21
Average	87 ^a,b^	21 ^a,b^	90	25	89	31

According to the consensus diagnosis, dermal mitoses are present in 28 cases. The table is intended to give an overview of the concordance and number of reported dermal mitoses over time and doesn’t show if the reported dermal mitoses are correct with respect to the consensus diagnosis.

**Table 2:** Kappa values (95% confidence interval) for all cases excluding nevoid melanoma (*n* = 89). ^a^ Glass; ^b^ WSI.

Pathologist	z-Stack Study Glass ^a^/WSI ^b^	1st Round WSI	2nd Round WSI Algorithm
EXP1	0.94 ^a^	0.89 (0.80–0.98)	0.83 (0.73–0.94)
EXP2	0.88 ^a^	0.83 (0.72–0.94)	0.79 (0.67–0.91)
PATH1	0.78 (0.66–0.90) ^b^	0.83 (0.72–0.94)	0.83 (0.72–0.94)
PATH2	0.79 (0.67–0.91) ^b^	0.92 (0.84–1.00)	0.89 (0.80–0.98)
PATH3	0.66 (0.51–0.79) ^b^	0.76 (0.64–0.88)	0.85 (0.75–0.95)
PATH4	0.55 (0.39–0.70) ^b^	0.70 (0.56–0.84)	0.55 (0.41–0.69)
PATH5	0.72 (0.58–0.83) ^b^	0.92 (0.84–1.00)	0.90 (0.81–0.98)
PATH6	0.81 (0.69–0.91) ^b^	0.73 (0.61–0.85)	0.73 (0.61–0.85)

**Table 3:** Concordance of nevoid melanoma (*n* = 10) as a percentage (%) of cases identical with the consensus diagnosis based on glass slides and number of cases with reported dermal mitosis (#DM).

Pathologist	z-Stack Study WSI		1st Round WSI		2nd Round WSI Algorithm	
	%	#DM	%	#DM	%	#DM
EXP1	-	-	70	7	70	5
EXP2	-	-	80	4	90	4
PATH1	50	4	70	6	80	8
PATH2	70	6	90	9	90	8
PATH3	20	0	40	3	70	3
PATH4	10	1	50	7	90	7
PATH5	80	4	70	5	70	6
PATH6	80	3	70	4	40	3
Average	52	3	68	5,6	75	5,5
